# Beyond nodes and edges: a bibliometric analysis on graph theory and neuroimaging modalities

**DOI:** 10.3389/fnins.2024.1373264

**Published:** 2024-04-23

**Authors:** Makliya Mamat, Ziyan Wang, Ling Jin, Kailong He, Lin Li, Yiyong Chen

**Affiliations:** ^1^School of Basic Medical Sciences, Health Science Center, Ningbo University, Ningbo, China; ^2^Department of Human Anatomy, Nanjing Medical University, Nanjing, China

**Keywords:** brain network, graph theory, neuroimaging, bibliometric analysis, research trends

## Abstract

Understanding the intricate architecture of the brain through the lens of graph theory and advanced neuroimaging techniques has become increasingly pivotal in unraveling the complexities of neural networks. This bibliometric analysis explores the evolving landscape of brain research by focusing on the intersection of graph theoretical approaches, neuroanatomy, and diverse neuroimaging modalities. A systematic search strategy was used that resulted in the retrieval of a comprehensive dataset of articles and reviews. Using CiteSpace and VOSviewer, a detailed scientometric analysis was conducted that revealed emerging trends, key research clusters, and influential contributions within this multidisciplinary domain. Our review highlights the growing synergy between graph theory methodologies and neuroimaging modalities, reflecting the evolving paradigms shaping our understanding of brain networks. This study offers comprehensive insight into brain network research, emphasizing growth patterns, pivotal contributions, and global collaborative networks, thus serving as a valuable resource for researchers and institutions navigating this interdisciplinary landscape.

## 1 Introduction

The unraveling of the brain’s intricate architecture stands as one of the most profound scientific endeavors, perpetually evolving to grasp the enigmatic complexity of the human brain (Tononi et al., 1994; Ramón Y Cajal, 1995; Bota et al., 2003). Understanding the brain’s organizational principles, connectivity patterns, and dynamic interplay between its components holds the key to comprehending cognition, behavior, and various neurological disorders (Bassett and Bullmore, 2009; Rubinov and Sporns, 2010; van den Heuvel and Hulshoff Pol, 2010).

Graph theory, a mathematical discipline that is concerned with the study of graphs or networks, has emerged as a foundational framework for modeling and analyzing the complex systems inherent in fields that include neuroscience (Ramnani et al., 2004; Bullmore and Bassett, 2011; Sporns, 2013a). Graph theory offers a systematic and quantitative means of characterizing the intricate structural and functional connectivity patterns that underlie neural circuits (Sporns, 2011; Sporns, 2013a). Graph theory facilitates a rigorous assessment of network properties, such as efficiency, resilience, and modularity, through the abstraction of the brain’s architecture into a network comprised of nodes and edges (Bassett and Bullmore, 2006; Rubinov and Sporns, 2010; Stam, 2014; Ma et al., 2021; Zamani Esfahlani et al., 2021). Nodes within the brain network typically correspond to anatomically or functionally defined brain regions, and edges represent the connections between them. These connections that can be derived from structural data (e.g., diffusion magnetic resonance imaging (MRI) tractography) or functional data (e.g., correlations in the function magnetic resonance imaging (fMRI) signal) encode a complex web of interactions that govern brain function (Watts and Strogatz, 1998).

Neuroimaging techniques provide a complementary path for probing the structure and function of the brain *in vivo*. These techniques encompass a spectrum of modalities, which range from traditional anatomical imaging methods that include structural MRI to advanced functional imaging methods such as resting-state fMRI and task-based fMRI (Ogawa et al., 1990; Conturo et al., 1999; Bandettini and Cox, 2000; Le Bihan and Johansen-Berg, 2012). Neuroimaging modalities yield rich datasets amenable to analysis within the framework of graph theory by capturing neural activity and connectivity patterns with high spatiotemporal resolution (Sporns et al., 2004; Hagmann et al., 2008; Honey et al., 2009).

The convergence of graph theory and neuroimaging has ushered in a transformative paradigm in the study of brain organization and function (Sporns, 2013b; Preti et al., 2017). This interdisciplinary synergy enables researchers to conceptualize the brain as a complex network. With the application of graph theoretical techniques to neuroimaging data, researchers can uncover fundamental principles that govern brain network organization, such as the presence of highly connected “hub” regions and the modular organization of functional brain networks (Crossley et al., 2014; Preti et al., 2017). Pioneering studies by Bullmore and Sporns (2009) and Bullmore and Sporns (2012) have elucidated the economy of brain network organization and complex brain networks’ structural and functional systems, respectively. Further, this integrative approach provides insight into how alterations in brain network topology relate to cognitive processes, behavior, and neurological disorders, thus advancing our understanding of brain function in health and disease (Menon, 2011; Fornito et al., 2012; Rubinov and Bullmore, 2013; Uddin et al., 2013; Iturria-Medina et al., 2014; Fornito et al., 2015).

Bibliometrics constitutes the analysis of published information and their associated metadata, such as abstracts, keywords, and citations. Bibliometrics aims to depict and elucidate relationships among these published works by employing statistical methods (Broadus, 1987; Hicks et al., 2015). This approach hinges on the premise that the scholarly output within a research domain is encapsulated within its published literature (Ninkov et al., 2022; Funada et al., 2023; Miao et al., 2023). The methodology of a bibliometric analysis encompasses diverse comprehensive techniques, including mathematical methods, network analyses, and clustering algorithms. These methodologies serve to scrutinize the overarching profiles of published works, thus presenting an objective and quantitative overview of the current status and evolving trends within various fields (Ying et al., 2023). In recent years, bibliometric analyses have gained widespread traction due to accessible software tools such as CiteSpace (Chaomei, 2006) and VOSviewer (van Eck and Waltman, 2010). The availability of these tools, coupled with the exponential growth in published literature, has rendered bibliometric analysis instrumental in comprehensively assessing the development trajectories of numerous specialized research fields (Brandt et al., 2019; Akmal et al., 2020; Hassan et al., 2021; Ge et al., 2022; Li et al., 2022).

In this study, we perform exhaustive systematic searches and rigorous data curation to meticulously compile a comprehensive dataset comprised of a multitude of articles and reviews to effectively capture the evolutionary trends within this expansive multidimensional domain. The integration of advanced visualization and data mining methodologies signifies a pioneering approach within bibliometric analyses, particularly for the exploration of brain networks through the amalgamation of graph theory and diverse neuroimaging techniques (van den Heuvel and Hulshoff Pol, 2010). This intersection represents a relatively uncharted territory that holds immense promise for unveiling novel insight into the complex landscape of brain connectivity.

Our primary objective is the elucidation of the intellectual trajectory and unfolding trends within the realm of brain network investigations over several decades. Our secondary goals is to provide a comprehensive evaluation of the diverse research networks that span across countries, institutions, authors, and journals. This holistic assessment approach allows us to delve deeper into collaborative networks, investigate key research productivity metrics, and pinpoint pivotal gaps within this dynamic interdisciplinary domain.

Further, our study seeks to identify prospective pathways, thus paving the way for potential directions and advancements in this multifaceted field. By critically evaluating collaborative networks and productivity metrics, our intent is not only to outline existing achievements, but also to identify crucial gaps that require further exploration and investigation. This multifaceted approach aims to significantly contribute to the understanding of brain network research dynamics, enabling the field to effectively navigate and chart future trajectories.

## 2 Materials and methods

### 2.1 Data collection

The data for the bibliometric analysis were obtained from the Clarivate Analytics’ Web of Science Core Collection (WOSCC), which included SCI-EXPANDED, SSCI, AHCI, ESCI, CCR-EXPANDED, IC, and search literature with the time span from January 1, 1995 through December 31, 2022. A bibliometric analysis of the retrieved documents was performed after searching in accordance with the abovementioned method. A total of 2,236 records, including 2,103 articles and 133 reviews were collected, and the records were exported in the “plain text” and “tab delimited file” format. Every document record included the title, author, keywords, abstract, year, organization, citation, and other relevant information.

### 2.2 Data analysis

**Figure 1:**
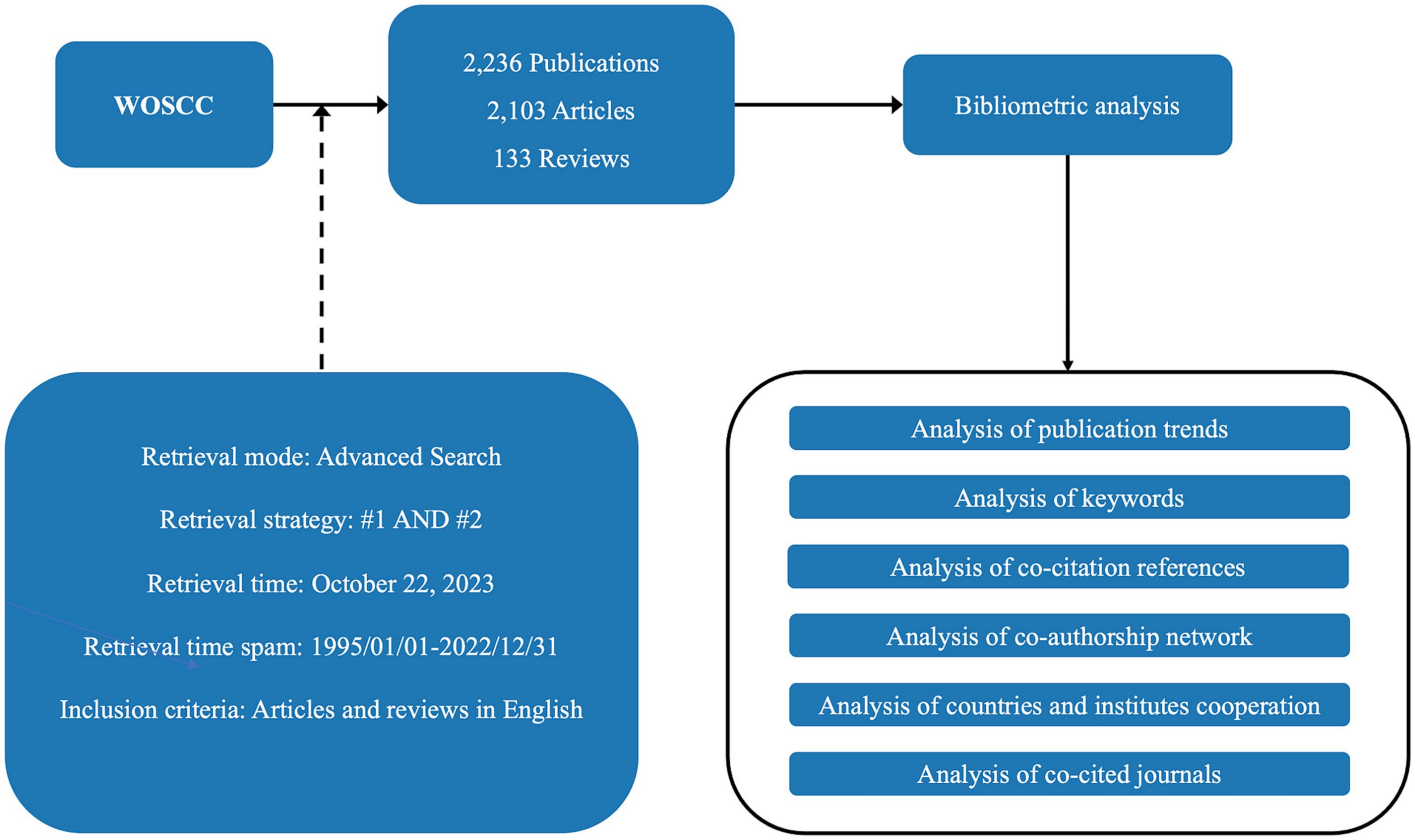
Flow chart of the study design. #1: [TS = (“Graph Theory” OR “Network Theory” OR “Network Analysis”) AND TS = (“brain” OR “Gray matter” OR “White matter” OR “Brain structure” OR “Brain anatomy” OR “brain connectivity” OR “Neural circuitry” OR “connectome”)]; #2: [TS = (“structural magnetic resonance imaging” OR “sMRI” OR “functional magnetic resonance imaging” OR “fMRI” OR “magnetic resonance spectroscopy” OR “MRS” OR “diffusion-tensor imaging” OR “DTI” OR “functional near-infrared spectroscopy” or “fNIRS” OR “single-photon emission computed tomography” OR “SPECT” OR “positron emission tomography” OR “PET”)]. WOSCC, web of science core collection.

VOSviewer 1.6.19 and CiteSpace 6.2.R5 were selected as the main bibliometric analysis tools to comprehensively analyze and summarize papers. To regulate the inclusion or exclusion of nodes in CiteSpace, the scale factor was adjusted to *k* = 25, the selection criteria was set to a top *N* = 50, and the time slice setting parameter was 2 years. Default configurations were maintained for all of the other settings (Chaomei, 2006). We conducted a cluster analysis and burst detection of keywords in order to understand the evolution of hotspots and predict the trends. Cluster labels were extracted from keyword lists using the log-likelihood ratio algorithm (*p* < 0.001). We identified the classical literature in the field using a co-citation analysis of the literature. Based on the contributions of countries, authors, and institutions, the cooperative co-occurrence graphs were drawn to analyze the connections between the elements. The flow chart of the study design is shown in Figure 1.

## 3 Results

### 3.1 Analysis of publication trends

**Figure 2:**
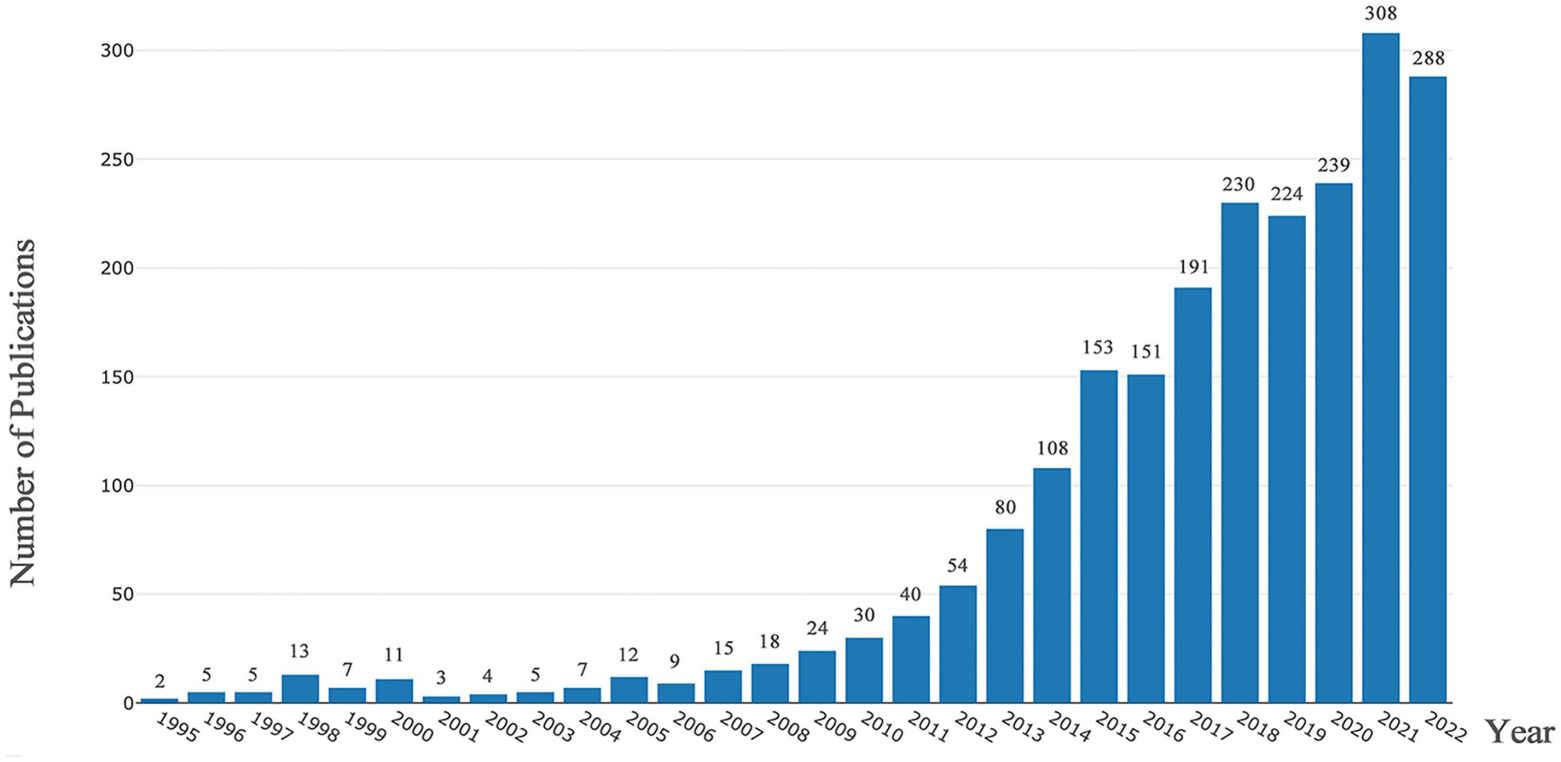
Number of annual research publications and growth trends from 1995 to 2022.

From a modest beginning in 1995, the field has witnessed remarkable and sustained growth in scholarly output. The publication count surged significantly in recent decades, thus indicating a pivotal turning point in the domain’s development. This acceleration continued unabated throughout the next decade, with each year consistently surpassing the previous one in terms of the number of articles published. The climax in 2021, with a record-breaking 308 articles, underlines the growing importance of this multidisciplinary field. Although 2022 showed a slight reduction in publication numbers, it is essential to consider it within the context of the field’s overall trajectory (Figure 2).

### 3.2 Keyword analysis

#### 3.2.1 Keyword network analysis

**Figure 3:**
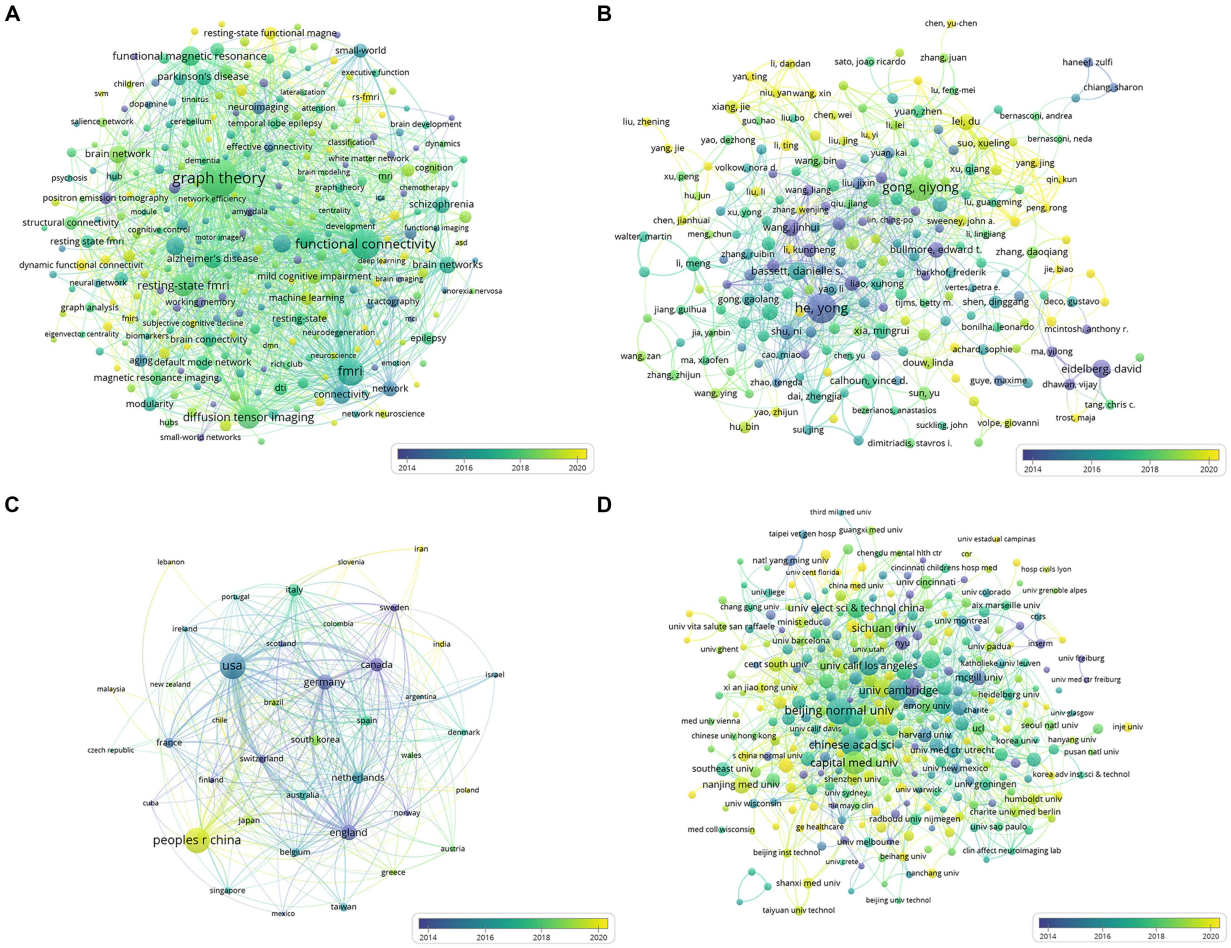
**(A)** Network of co-occurring author keywords; **(B)** Network of cooperation between authors; **(C)** Network of cooperation between countries/regions; **(D)** Network of cooperation between institutions. The size of a node is proportional to the frequency of its occurrence. The color of the node corresponds to the average year of publication.

A total of 2,236 documents were examined, and a compilation of 287 author-generated keywords offered insight into the prevailing themes and research directions within this field. VOSviewer was used to produce an overlay visualization based on the average publication year (Figure 3A) to delineate keyword co-occurrences. The top 10 keywords, ranked by frequency, encapsulated the focal points of the current research endeavors: ‘graph theory,’ ‘functional connectivity,’ ‘fMRI,’ ‘connectivity,’ ‘organization,’ ‘brain networks,’ ‘resting-state fMRI,’ ‘cortex,’ ‘small-world,’ and ‘MRI’ (Supplementary Table S1). This analysis illuminated the thematic emphasis and prevalent areas of exploration within brain network research, outlining the dominant concepts and directions that have garnered significant attention among researchers.

#### 3.2.2 Keywords citation burst analysis

A burst analysis was performed to identify the keywords that exhibited the most dynamic evolution over time. The top three keywords that demonstrated the strongest burst strength were ‘network analysis,’ ‘positron emission tomography,’ and ‘human brain.’ These keywords signified areas that have experienced significant surges in interest and focus within the literature.

Further, the keywords ‘significant difference’ and ‘machine learning’ displayed recent bursts, showing heightened activity specifically from 2020 to 2022 (Supplementary Table S2). This temporal analysis highlighted emerging areas of interest and reflected the evolving landscape within brain network research, indicating noteworthy shifts in attention and emphasis within the field during this period.

#### 3.2.3 Keywords time zone map analysis

The time zone map for keywords visually depicts the temporal evolution of high-frequency keywords within the context of their appearance over time. This representation provides a clear visualization of the emergence periods of these keywords, enabling an understanding of their hotspots and potential future trends.

In this visualization, larger circles indicate keywords cited more frequently, reflecting heightened discussions and increased research activity around those specific terms. Supplementary Figure S1 shows a comprehensive overview of the prominence and temporal distribution of these keywords, serving as a valuable tool to identify the historical emergence and current significance of these terms within brain network research.

### 3.3 Analysis of co-citation references

#### 3.3.1 Cluster network of research

**Figure 4:**
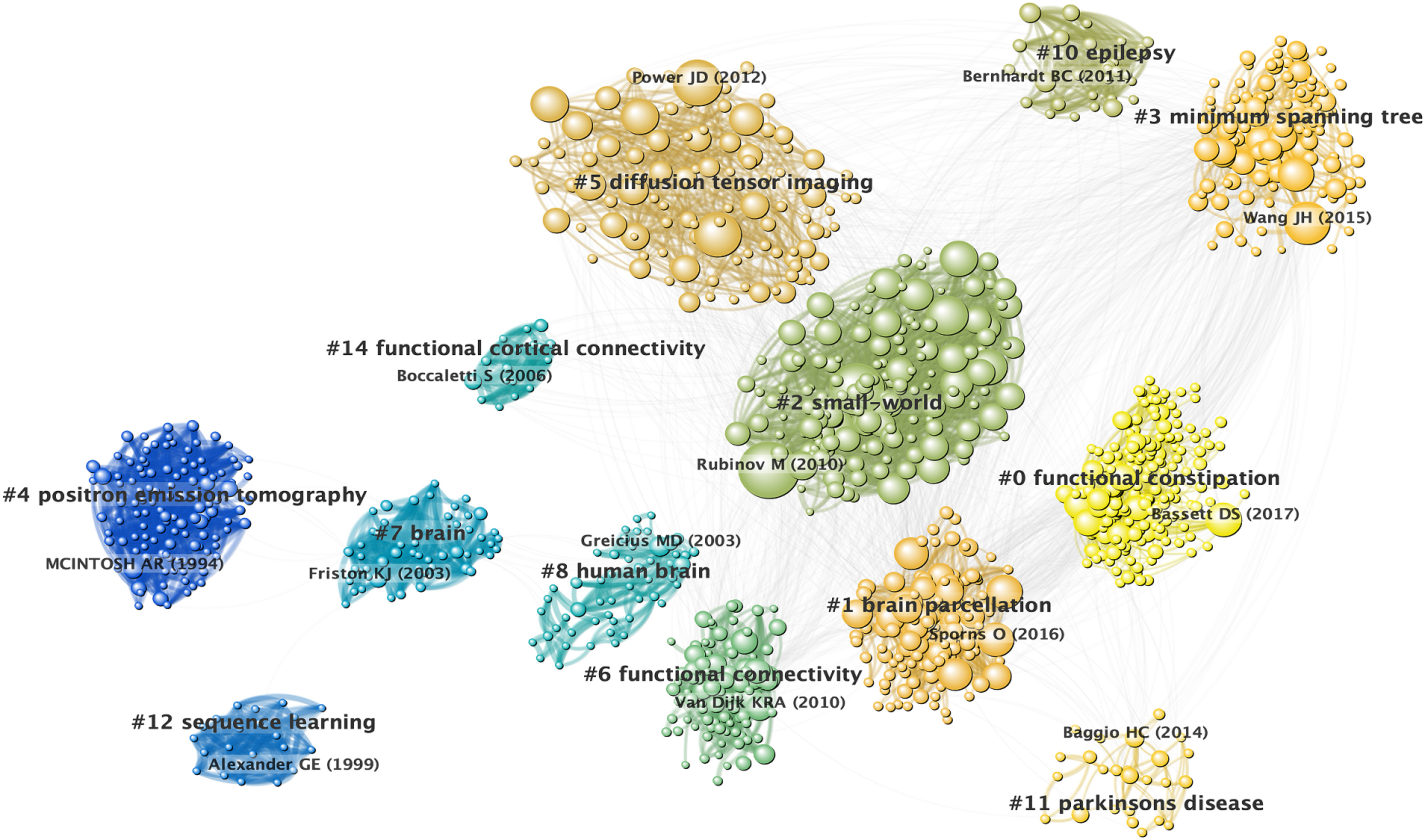
Co-citation references network and corresponding clustering visualization. A node represents a cited reference. The size of a node is proportional to its citation.

An analysis of the co-citation references revealed a rich landscape comprised of 23 distinct clusters that emphasized significant modularity (*Q* = 0.6946) and high silhouette scores (S = 0.879). These metrics underscored the reliability and distinctiveness of the identified clusters, with further scrutiny focused on 15 of these clusters for visualization within the co-citation references network (Figure 4). For detailed insights into each cluster’s thematic context, comprehensive descriptions are provided in Supplementary Table S3.

Each cluster exhibited a particularly high silhouette score indicative of a well-defined and distinct research focus within its domain. This comprehensive clustering analysis not only reinforced the reliability of the identified thematic clusters, but also offered a nuanced understanding of the diverse research avenues prevalent within brain network investigations.

#### 3.3.2 Co-cited references timeline map

**Figure 5:**
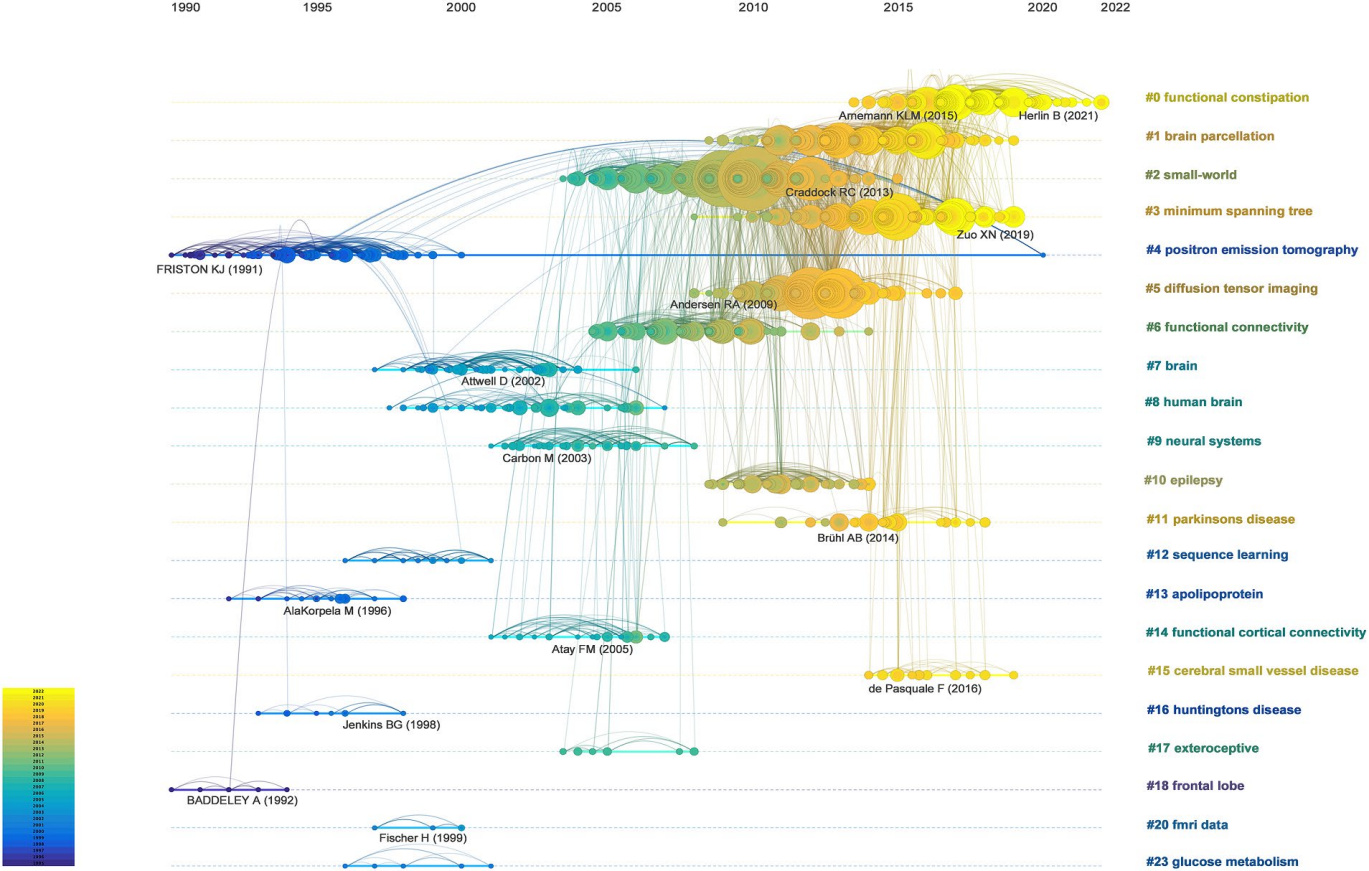
Timeline visualization of co-citation references network. A node represents a cited reference. For each cluster, nodes are organized by their year of publication on horizontal lines. The color of lines indicate the time of links between nodes or between clusters.

The timeline map of the co-cited references was constructed by labeling the clusters using noun terms extracted from keywords. The arrangement of the node centers along the horizontal axis from left to right signifies the initial publication year of the cited documents, capturing the temporal evolution of the literature. This layout offered insights into the temporal characteristics and evolutionary trends of the referenced literature (Figure 5).

Certain study topics within the timeline map exhibited shorter durations, such as cluster (#12) that focuses on sequence learning. Conversely, other clusters, namely (#0) functional constipation and (#1) brain parcellation, continued to demonstrate ongoing activity, highlighting persistent research hotspots within this field. This visualization enables the identification of both transient and enduring research trends, providing a comprehensive temporal perspective on the evolution of cited literature and highlighting focal areas of ongoing interest and investigation within brain network research.

#### 3.3.3 Most cited references and high centrality papers

We meticulously identified the top 10 most cited references within the field, representing pivotal works that offer substantial insights into the subject matter. The comprehensive list of these references is presented in Table 1, signifying their significant contributions to the field’s advancement. One study has garnered exceptional attention and recognition: ‘Complex Network Measures of Brain Connectivity: Uses and Interpretations’ authored by Rubinov and Sporns (2010), and it stands out as the most frequently cited reference, having amassed an impressive citation count of 201.

**Table 1:** The top 10 most cited references.

**Number of citations in the network / literature (October 2023)**	**Year**	**Title**	**Source**	**DOI**	**Cluster ID**
201/10585	2010	Complex network measures of brain connectivity: uses and interpretations	Neuroimage	10.1016/j.neuroimage.2009.10.003	#2
161/11712	2009	Complex brain networks: graph theoretical analysis of structural and functional systems	Nat Rev Neurosci	10.1038/nrn2575	#2
132/6835	2012	Spurious but systematic correlations in functional connectivity MRI networks arise from subject motion	Neuroimage	10.1016/j.neuroimage.2011.10.018	#5
124/3393	2013	BrainNet Viewer: a network visualization tool for human brain connectomics	Plos One	10.1371/journal.pone.0068910	#5
111/1082	2015	GRETNA: a graph theoretical network analysis toolbox for imaging connectomics	Front hum Neurosci	10.3389/fnhum.2015.00386	#3
95/3171	2012	The economy of brain network organization	Nat Rev Neurosci	10.1038/nrn3214	#2
75/4520	2008	Mapping the structural core of human cerebral cortex	Plos Biol	10.1371/journal.pbio.0060159	#2
75/2904	2009	Cortical hubs revealed by intrinsic functional connectivity: mapping, assessment of stability, and relation to Alzheimer’s disease	J Neurosci	10.1523/JNEUROSCI.5062-08.2009	#2
74/1956	2013	Network hubs in the human brain	Trends Cogn Sci	10.1016/j.tics.2013.09.012	#5
73/2379	2012	The influence of head motion on intrinsic functional connectivity MRI	Neuroimage	10.1016/j.neuroimage.2011.07.044	#5

Among these top references, three papers exhibited higher centrality, indicating their substantial influence on the field’s trajectory (Supplementary Table S4). These included ‘A Network Analysis of the Default Mode Hypothesis’ by Greicius et al. (2003), offering evidence for the cohesive existence of the default mode network; a comprehensive review by McIntosh (1999), elucidating neural systems and their correlation with cognition; and a clinical trial conducted by McIntosh et al. (1996), revealing changes in limbic and prefrontal functional interactions. These papers, distinguished by their higher centrality, underscore their profound impact and influential contributions within the domain of brain network research.

### 3.4 Analysis of co-authorship networks

A network analysis of the co-cited authors revealed substantial modularity and silhouette scores (*Q* = 5,004; *S* = 0.7459), as depicted in Supplementary Figure S2. Among the clusters identified, Cluster #0, titled ‘Diffusion Tensor Imaging,’ emerged as the most significant and central cluster, emphasizing its pivotal role within the network. Supplementary Table S5 provides detailed descriptions for each cluster, offering comprehensive insights into their thematic focus and significance.

The top three most cited authors within this network were Rubinov M (*n* = 1,101 citations), Bullmore ET (*n* = 995), and Sporns O (*n* = 734), showcasing their significant contributions to the field. Further, the top three authors with the strongest betweenness centrality, indicating their pivotal role in connecting different segments of the network, were Friston KJ (0.08), Horwitz (0.07), and Raichle ME (0.05), as outlined in Supplementary Table S6. McIntosh AR emerged as the author with the strongest burst strength, indicating a notable surge in citations and an active contribution from 1995 to 2010, detailed in Supplementary Table S7.

Further, an analysis of the collaborative network of citing authors delineated influential cooperative groups, prominently led by Gong QY, He Y, Lei D, Suo XL, and Shu N (Figure 3B). These authors played instrumental roles within the network, significantly contributing to collaborative efforts and advancing research in the field, showcasing the importance of collaborative endeavors in shaping the landscape of brain network research.

### 3.5 Analysis of co-cited journals

An analysis of the co-cited journals highlighted the top three most cited publications: Neuroimage (*n* = 2,146 citations), Human Brain Mapping (*n* = 1787), and the Journal of Neuroscience (*n* = 1,657), demonstrating their significant influence within neuroimaging and neuroscience research. These journals have served as pivotal platforms for disseminating cutting-edge research in the field.

Further, the top three journals with the highest centrality, signifying their critical role in connecting various segments of the network, were Experimental Brain Research (0.05), Archives of Neurology (0.04), and Alzheimer Disease and Associated Disorders (0.04), as detailed in Supplementary Table S6. These journals have played instrumental roles in fostering the exchange and dissemination of neuroimaging and brain graph analysis research, contributing significantly to the field’s advancement.

It is worthy to note that NeuroReport emerged as the journal with the strongest burst, signaling a substantial increase in citations and continued activity from 1995 to 2012. Further, recent surges were observed in Scientific Reports, Nature Communications, Network Neuroscience, Frontiers in Aging Neuroscience, and Dialogues in Clinical Neuroscience, as indicated in Supplementary Table S8. These journals represent recent avenues of prominence, offering potential outlets for researchers engaged in neuroimaging and graph analysis to consider for their publications.

### 3.6 Analysis of countries and institutes cooperation

Figures 3C,D illustrate the cooperative networks among countries and institutions, respectively. Our analysis encompassed 41 countries or regions. The top three countries that contributed the most papers were the United States (873 papers, 39.04%), the People’s Republic of China (812 papers, 36.31%), and England (212 papers, 9.48%). In terms of citations, the United States was the most cited country (*n* = 62,156), followed by England (*n* = 25,325) and the People’s Republic of China (*n* = 22,970), as detailed in Supplementary Table S8.

Moreover, our dataset encompassed 322 institutions. Beijing Normal University emerged as the institution with the highest number of publications (*n* = 114), followed by Capital Medical University (*n* = 85) and the Chinese Academy of Sciences (*n* = 80). In terms of citations, Beijing Normal University secured the second position (*n* = 8,853), with the University of Cambridge leading the citations (*n* = 15,643), as depicted in Supplementary Table S9.

## 4 Discussion

Our study used an exhaustive bibliometric analysis to provide a comprehensive overview of brain network research dynamics. We highlighted the transformative synergy between graph theory and neuroimaging techniques, revealing diverse research clusters and showcasing the influential roles of countries, institutions, authors, and journals. Our findings underscored the multidimensional nature of contemporary investigations and the collaborative networks that drive advancements in this dynamic field.

The integration of graph theory methodologies with advanced neuroimaging techniques represents a pivotal advancement in neuroscience, fundamentally transforming our understanding of brain networks (Hagmann et al., 2008; Park and Friston, 2013; Bassett and Bullmore, 2017; Breakspear, 2017). Graph theory offers a robust mathematical framework for modeling complex relationships within brain networks, providing insight into their organizational principles (Watts and Strogatz, 1998; Bullmore and Sporns, 2009). When paired with cutting-edge neuroimaging modalities, such as fMRI and diffusion tensor imaging (DTI), this integration empowers researchers to visualize, analyze, and comprehend the brain’s intricate connectivity patterns *in vivo* (Biswal et al., 2010; Bullmore and Bassett, 2011; Deco et al., 2011; Le Bihan and Johansen-Berg, 2012; Sporns, 2013b). Studies by van den Heuvel and Sporns (2013) showcase how graph-based analyses unveil topological properties, small-world architectures, and decipher critical network hubs for information processing (Hagmann et al., 2008). He and Evans (2010) and Bullmore and Sporns (2012) emphasized that this integration transcends traditional neuroanatomical boundaries, fostering a holistic network-centric perspective. Such transformative integration, as outlined by researchers, has reshaped our understanding and shed light on how dynamic interactions between brain regions underpin cognitive processes, behavior, and neurological disorders (Bressler and Menon, 2010; Menon, 2011; Fornito et al., 2015; Bassett and Sporns, 2017).

Our comprehensive analysis unveiled dynamic trends and distinctive clusters that delineated the multifaceted landscape of brain network research. Among the identified clusters, sustained areas, such as functional connectivity and specific brain parcellation, emerged as enduring focal points. This was substantiated by their consistent activity and extensive exploration over time. For instance, functional constipation, that exhibited sustained prominence, signified its pivotal role in understanding the brain’s functional dynamics, garnering ongoing interest and exploration (Duan et al., 2021; Liu et al., 2021; Peihong et al., 2021; Yu et al., 2023). Conversely, emerging clusters, including sequence learning, showcased the field’s adaptability by embracing novel areas of investigation. Sequence learning’s recent surge in scholarly attention implies its potential for unraveling the brain’s cognitive mechanisms, and it is an evolving area of interest that will likely bring future breakthroughs (Yeo et al., 2011; Watanabe et al., 2019). These sustained and emerging clusters not only signify the depth and breadth of research in established domains, but also hint at the field’s receptiveness to novel methodologies, fostering continuous innovation and exploration within brain network investigations.

Further, recent advancements in neuroimaging technology have propelled the integration of machine learning methodologies into brain network analyses. Graph neural networks, for example, have demonstrated remarkable efficacy in capturing the intricate connectivity patterns within functional brain networks (Li et al., 2021). These networks offer a powerful framework for modeling brain dynamics by leveraging graph structures to represent complex relationships between brain regions. In our analysis, keywords such as “machine learning” and “deep learning” emerged (Figure 3A; Supplementary Figure S1; Supplementary Table S2), reaffirming the growing relevance of these methodologies in the realm of brain network research. This convergence of graph theory and machine learning holds significant promise for unraveling the intricacies of brain connectivity, paving the way for innovative approaches to understanding brain function and dysfunction.

The vibrant collaborative networks that span countries, institutions, authors, and journals underscore the dynamic interplay that molds brain network research. The strategic collaborations between countries such as the United States, the People’s Republic of China, and England signify a global alliance driving advancements in this field, highlighting the significance of international partnerships in accelerating research progress. Key institutions, such as the Beijing Normal University and the University of Cambridge, play pivotal roles, demonstrating their influential contributions in steering research trajectories and fostering cross-disciplinary investigations. Influential authors, exemplified by Rubinov and Bullmore (2013), not only spearhead collaborative networks, but they also contribute seminal works that shape the discourse within the field. Further, foundational references like Rubinov and Sporns (2010) work on complex network measures of brain connectivity stand as pillars in the field, amassing high citations and significantly influencing subsequent research directions. Esteemed journals, including Neuroimage, Human Brain Mapping, and the Journal of Neuroscience, serve as vital platforms for disseminating cutting-edge research, further solidifying their roles in propelling brain network investigations. These collaborative efforts, spanning diverse domains within brain network research, underscore the synergistic nature of collective contributions, affirming interdisciplinary growth and evolution within this dynamic field.

While our study provided valuable insights, certain limitations and gaps warrant consideration for future research endeavors. One notable limitation lies in the reliance on published literature, potentially overlooking unpublished or emerging research that could offer novel perspectives. The time span of our study may not have fully captured the most recent developments in the field. In addition, the scope of our analysis might have inadvertently omitted niche research or specific methodologies that could contribute substantially to the broader understanding of brain networks. Addressing these limitations could enrich future analyses and provide a more comprehensive view of the evolving landscape in this field.

### 4.1 Future directions

Moving forward, the findings of this study suggested several promising paths for future investigations in brain network research. First, delving deeper into the functional implications of sustained clusters could unveil underlying mechanisms that drive brain functionality. Second, the exploration of emergent clusters is an exciting opportunity to uncover novel cognitive mechanisms. Further, the incorporation of interdisciplinary approaches that amalgamate graph theory with emerging neuroimaging techniques might offer fresh insight into brain connectivity. In addition, investigating the impact of neurological disorders on brain networks and exploring methodologies to characterize alterations in these conditions could pave the way for diagnostic and therapeutic advancements. Future studies could focus on fostering international collaborations to facilitate data sharing and standardization, fostering a more unified understanding of brain network dynamics across diverse populations. These proposed directions aim to bridge existing gaps, stimulate innovation, and further unravel the complexities of brain networks.

## 5 Conclusion

In summary, our comprehensive bibliometric analysis offers multifaceted insights into the intricate dynamics of brain network research. By elucidating evolving trends, collaborative networks, and key research clusters, our study underscored the transformative integration of graph theory with neuroimaging techniques, reshaping our understanding of brain connectivity. The sustained and emerging clusters identified revealed lasting areas of interest and indicated paths for future exploration, while collaborative networks among countries, institutions, authors, and journals highlighted the combined endeavors that can propel progress in this vibrant field. Importantly, the findings of this study serve as a foundation for future investigations, offering a panoramic view of brain network research dynamics. The implications of this study lie in its potential to guide future research directions, foster interdisciplinary collaborations, and inspire innovative methodologies, thus contributing significantly to unraveling the complexities of brain networks and advancing our understanding of cognitive processes, behaviors, and neurological disorders.

## Author contributions

MM: Data curation, Formal analysis, Resources, Software, Visualization, Writing – original draft. ZW: Data curation, Resources, Software, Writing – review & editing. LJ: Data curation, Software, Visualization, Writing – review & editing. KH: Resources, Software, Visualization, Writing – review & editing. LL: Conceptualization, Investigation, Methodology, Project administration, Supervision, Writing – review & editing. YC: Conceptualization, Investigation, Methodology, Supervision, Validation, Writing – review & editing.
