# Corrigendum: Beyond nodes and edges: a bibliometric analysis on graph theory and neuroimaging modalities

**DOI:** 10.3389/fnins.2024.1452045

**Published:** 2024-07-03

**Authors:** Makliya Mamat, Ziyan Wang, Ling Jin, Kailong He, Lin Li, Yiyong Chen

**Affiliations:** ^1^School of Basic Medical Sciences, Health Science Center, Ningbo University, Ningbo, China; ^2^Department of Human Anatomy, Nanjing Medical University, Nanjing, China

**Keywords:** brain network, graph theory, neuroimaging, bibliometric analysis, research trends

In the published article, there was an error in the legend for Figure 3 as published. Figure 3C includes regions in addition to countries. The corrected legend appears below.

“**(A)** Network of co-occurring author keywords; **(B)** Network of cooperation between authors; **(C)** Network of cooperation between countries/regions; **(D)** Network of cooperation between institutions. The size of a node is proportional to the frequency of its occurrence. The color of the node corresponds to the average year of publication.”

In the published article, there was also an error in Results, *3.6 Analysis of countries and institutes cooperation*, Paragraph 1. “Network of cooperation between countries” should be stated as “Network of cooperation between countries/regions”. A correction has been made to the paragraph:

“Figures 3C, D illustrate the cooperative networks among countries and institutions, respectively. Our analysis encompassed 41 countries or regions. The top three countries that contributed the most papers were the United States (873 papers, 39.04%), the People's Republic of China (812 papers, 36.31%), and England (212 papers, 9.48%). In terms of citations, the United States was the most cited country (*n* = 62,156), followed by England (*n* = 25,325) and the People's Republic of China (*n* = 22,970), as detailed in Supplementary Table S8.”

The authors apologize for these errors and state that this does not change the scientific conclusions of the article in any way. The original article has been updated.

