# Supplementary material for: Beyond nodes and edges: a bibliometric analysis on graph theory and neuroimaging modalities
Source: Front Neurosci. 2024 Apr 23;18:1373264. doi: 10.3389/fnins.2024.1373264 (PMC11074400; doi:10.3389/fnins.2024.1373264)
Supplement: Supplementary file 1 [file Presentation_1.PPTX]

## Slide 1
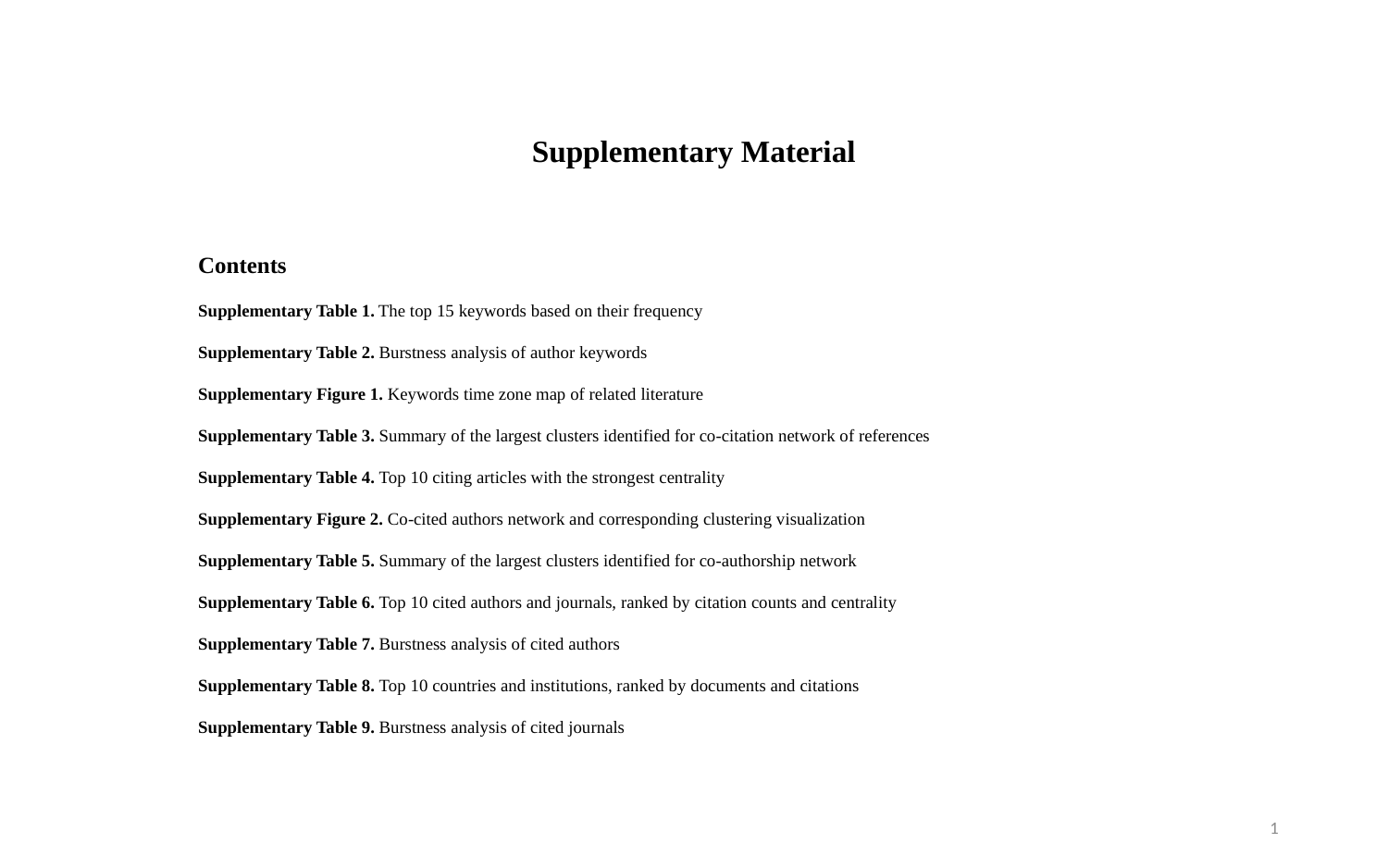

Supplementary Material
Contents
Supplementary Table 1. The top 15 keywords based on their frequency
Supplementary Table 2. Burstness analysis of author keywords
Supplementary Figure 1. Keywords time zone map of related literature
Supplementary Table 3. Summary of the largest clusters identified for co-citation network of references
Supplementary Table 4. Top 10 citing articles with the strongest centrality
Supplementary Figure 2. Co-cited authors network and corresponding clustering visualization
Supplementary Table 5. Summary of the largest clusters identified for co-authorship network
Supplementary Table 6. Top 10 cited authors and journals, ranked by citation counts and centrality
Supplementary Table 7. Burstness analysis of cited authors
Supplementary Table 8. Top 10 countries and institutions, ranked by documents and citations
Supplementary Table 9. Burstness analysis of cited journals
1

## Slide 2
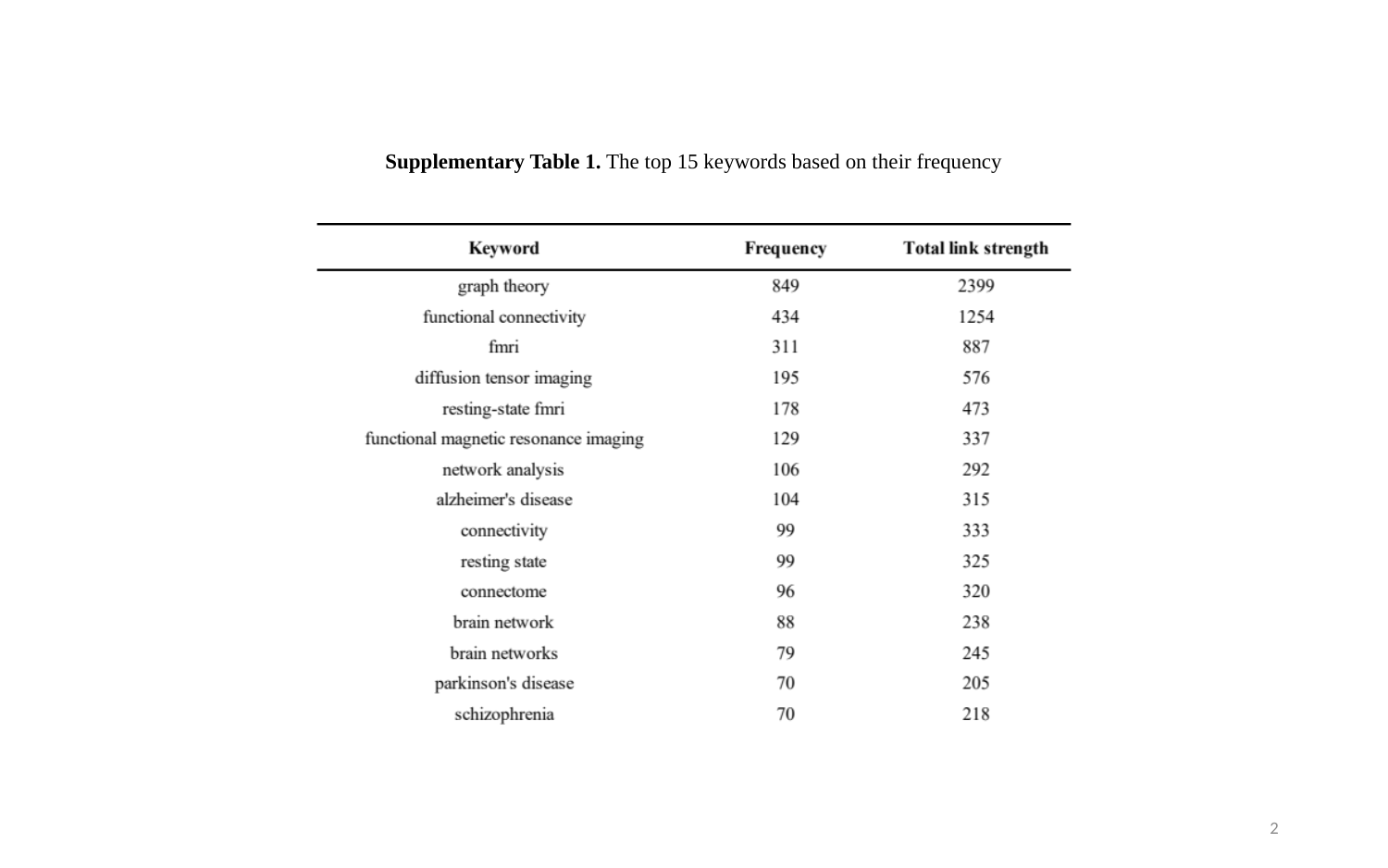

Supplementary Table 1. The top 15 keywords based on their frequency
2

## Slide 3
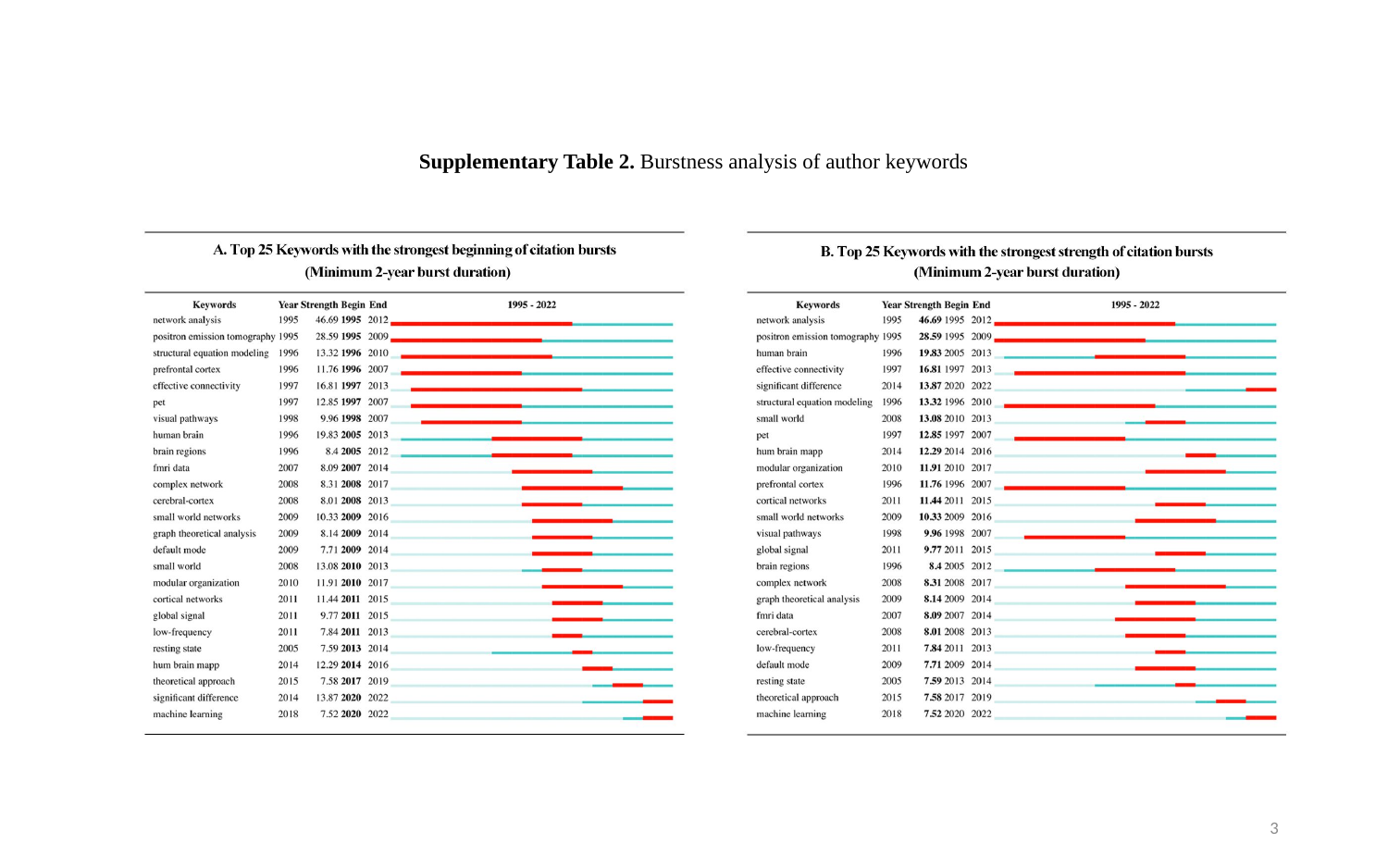

Supplementary Table 2. Burstness analysis of author keywords
3

## Slide 4
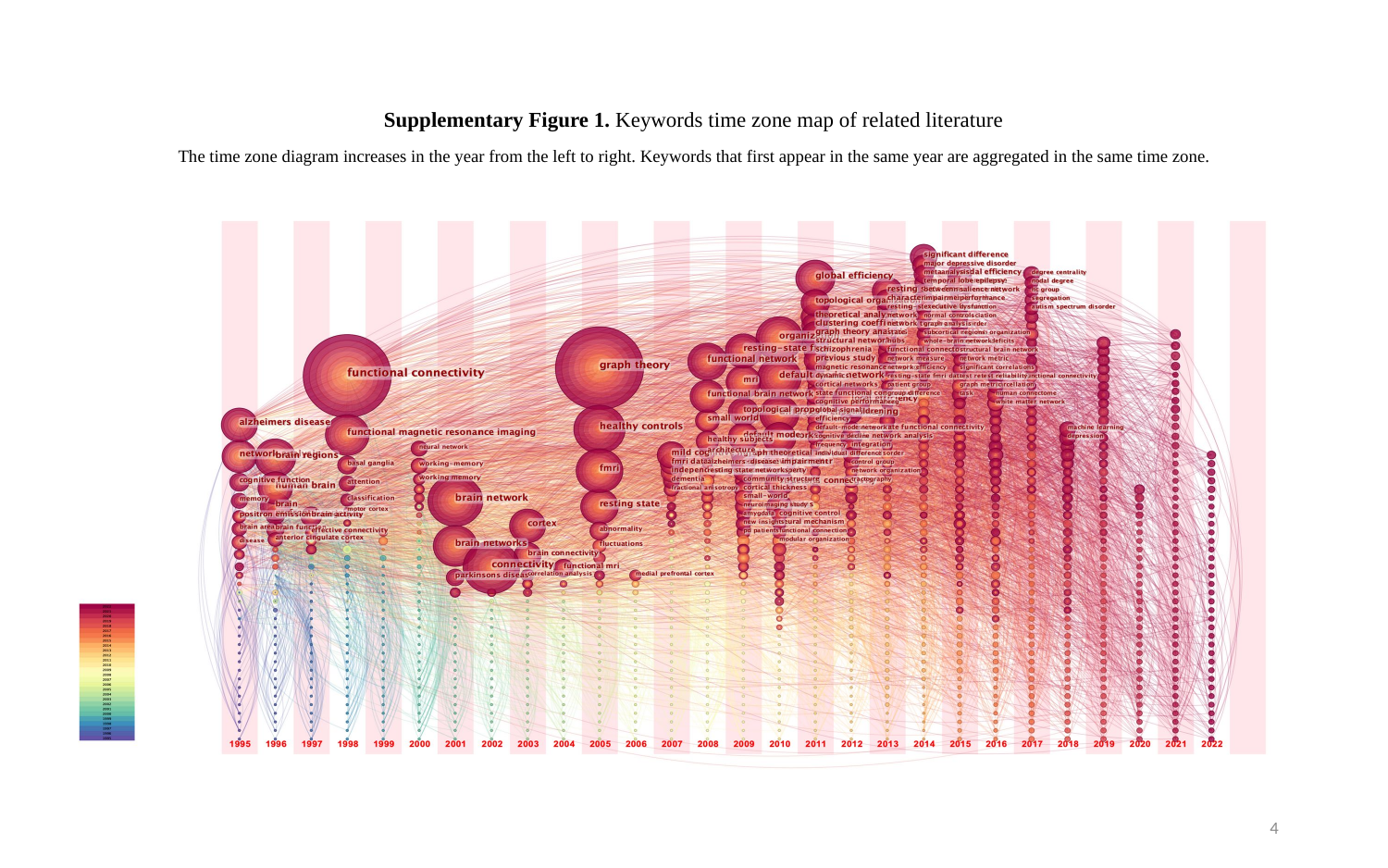

Supplementary Figure 1. Keywords time zone map of related literature
The time zone diagram increases in the year from the left to right. Keywords that first appear in the same year are aggregated in the same time zone.
4

## Slide 5
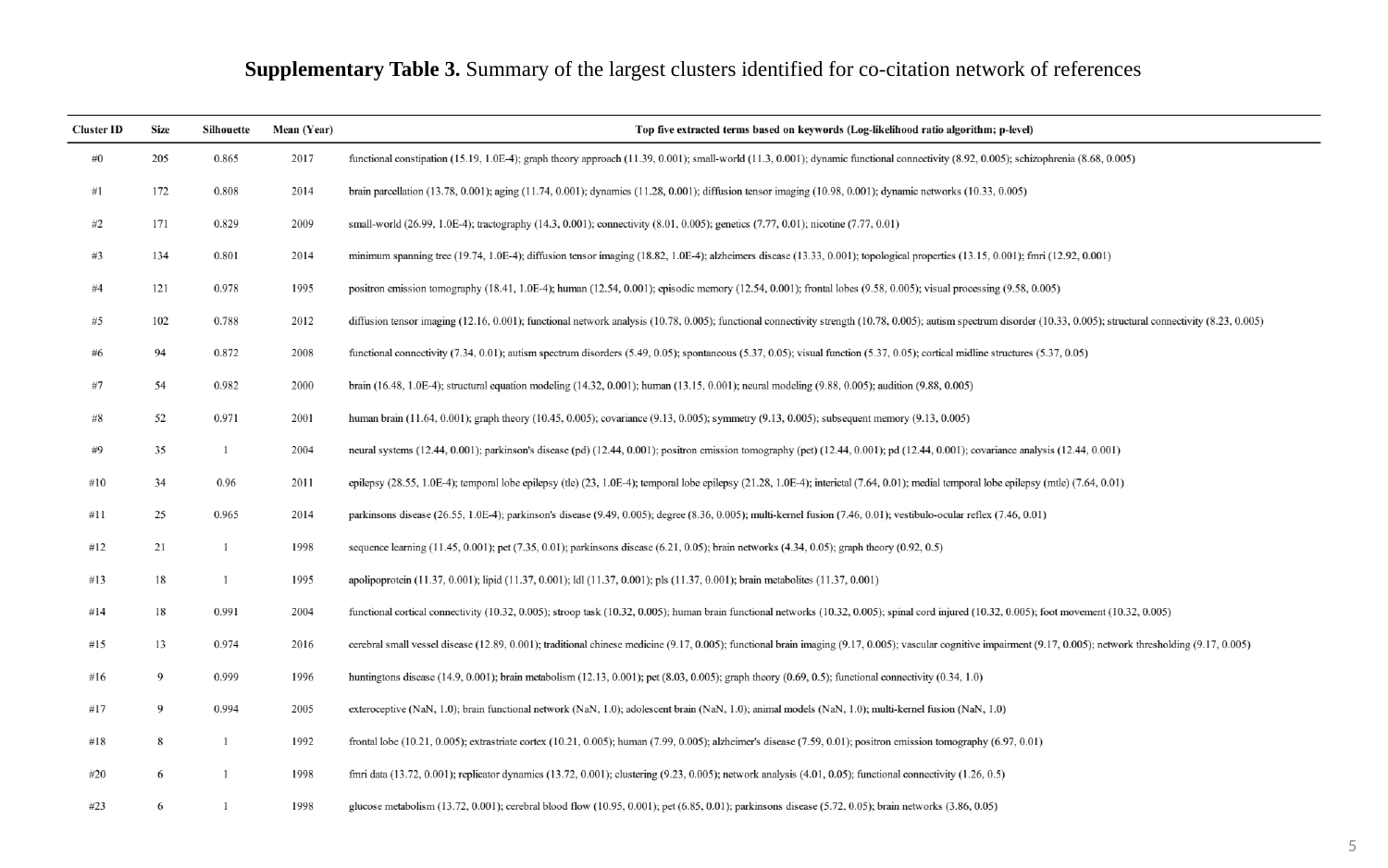

Supplementary Table 3. Summary of the largest clusters identified for co-citation network of references
5

## Slide 6
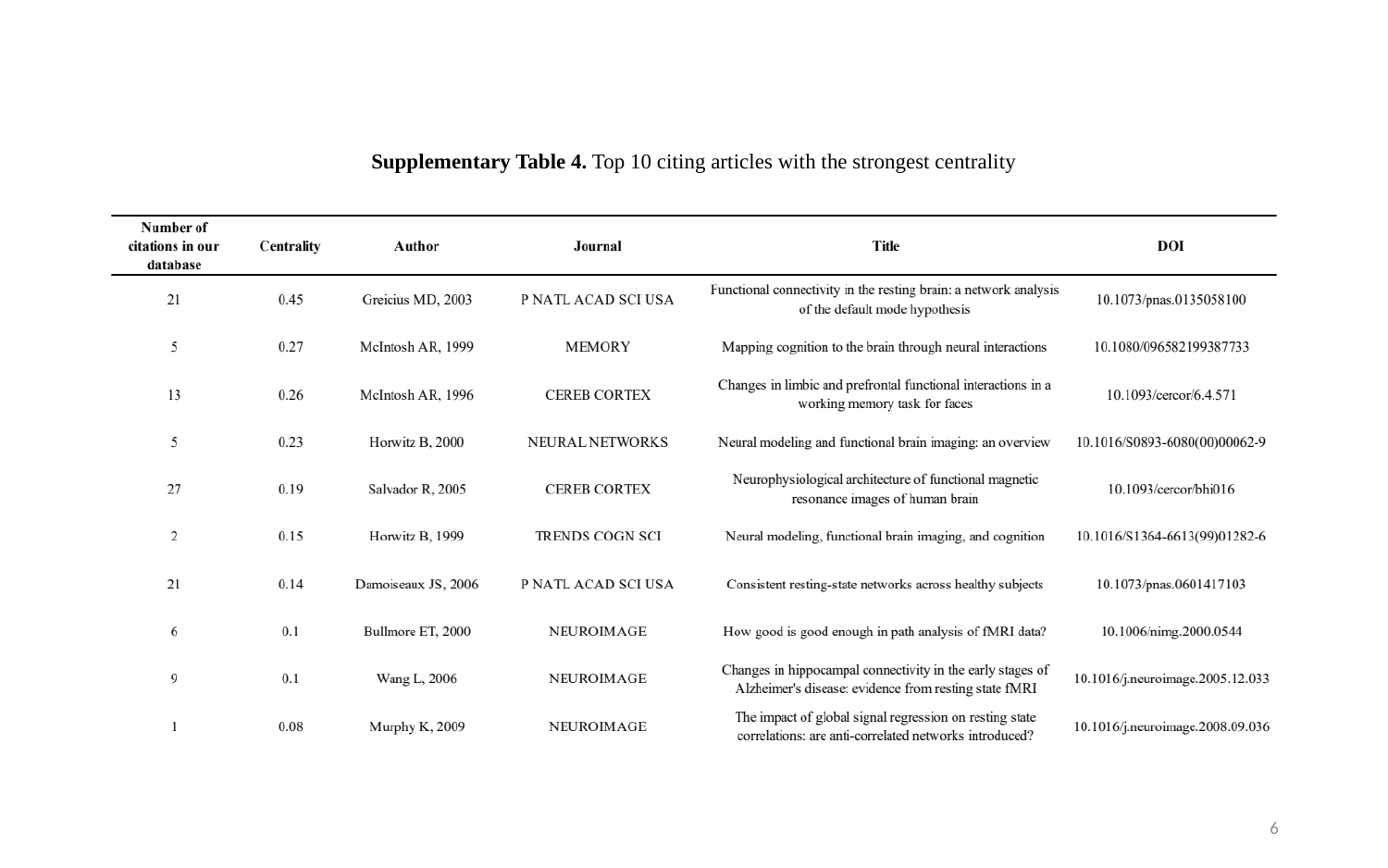

Supplementary Table 4. Top 10 citing articles with the strongest centrality
6

## Slide 7
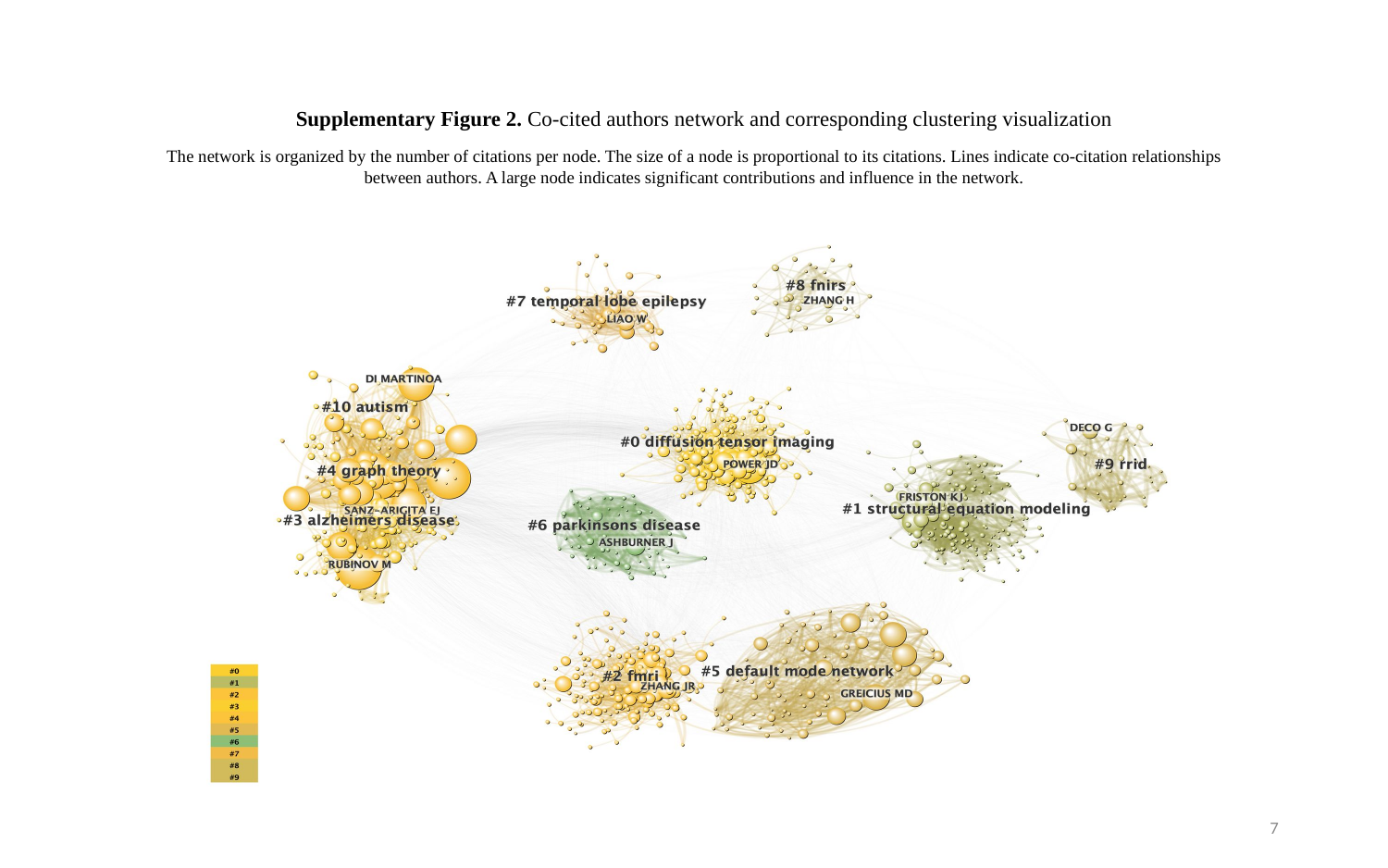

Supplementary Figure 2. Co-cited authors network and corresponding clustering visualization
The network is organized by the number of citations per node. The size of a node is proportional to its citations. Lines indicate co-citation relationships between authors. A large node indicates significant contributions and influence in the network.
7

## Slide 8
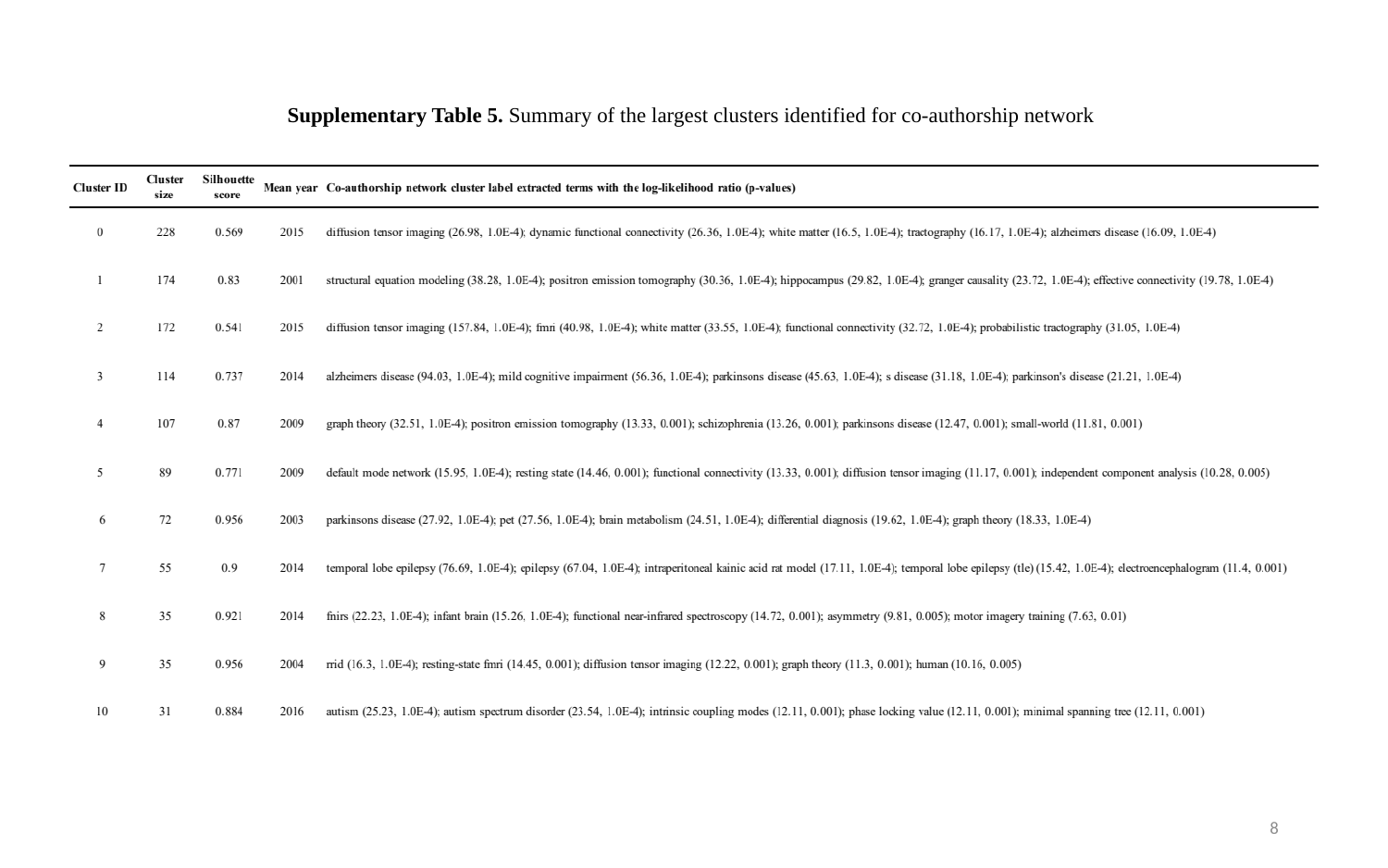

Supplementary Table 5. Summary of the largest clusters identified for co-authorship network
8

## Slide 9
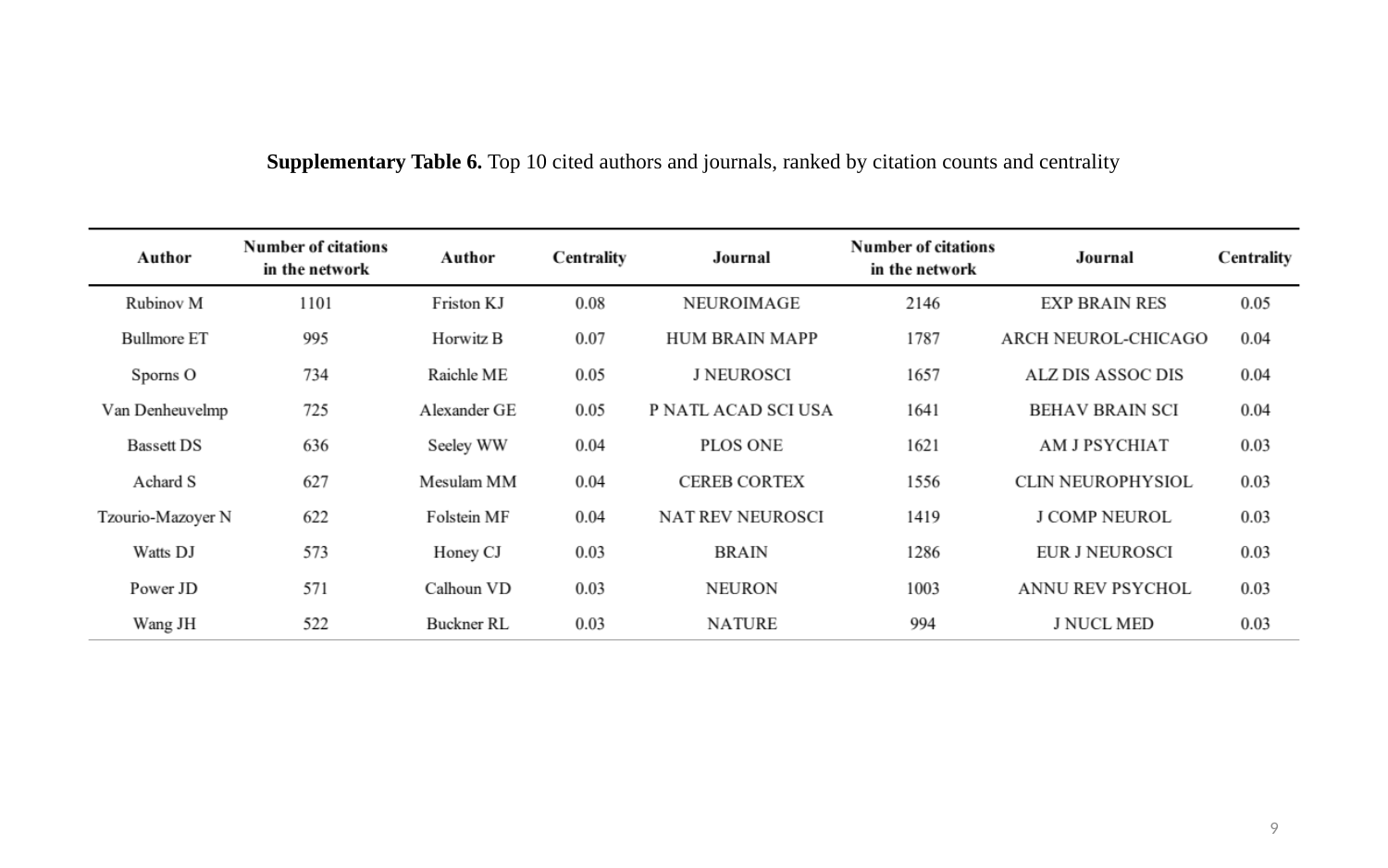

Supplementary Table 6. Top 10 cited authors and journals, ranked by citation counts and centrality
9

## Slide 10
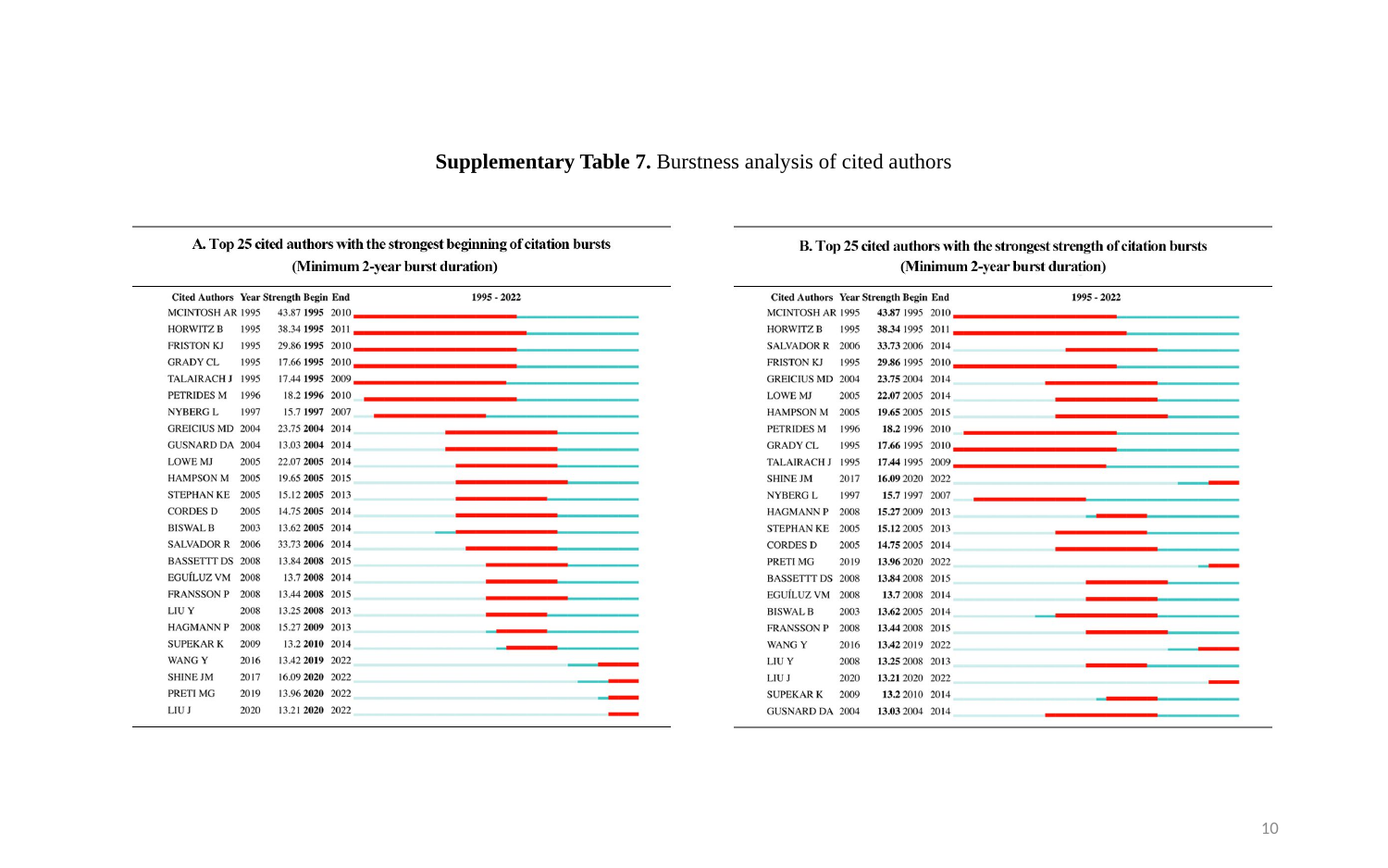

Supplementary Table 7. Burstness analysis of cited authors
10

## Slide 11
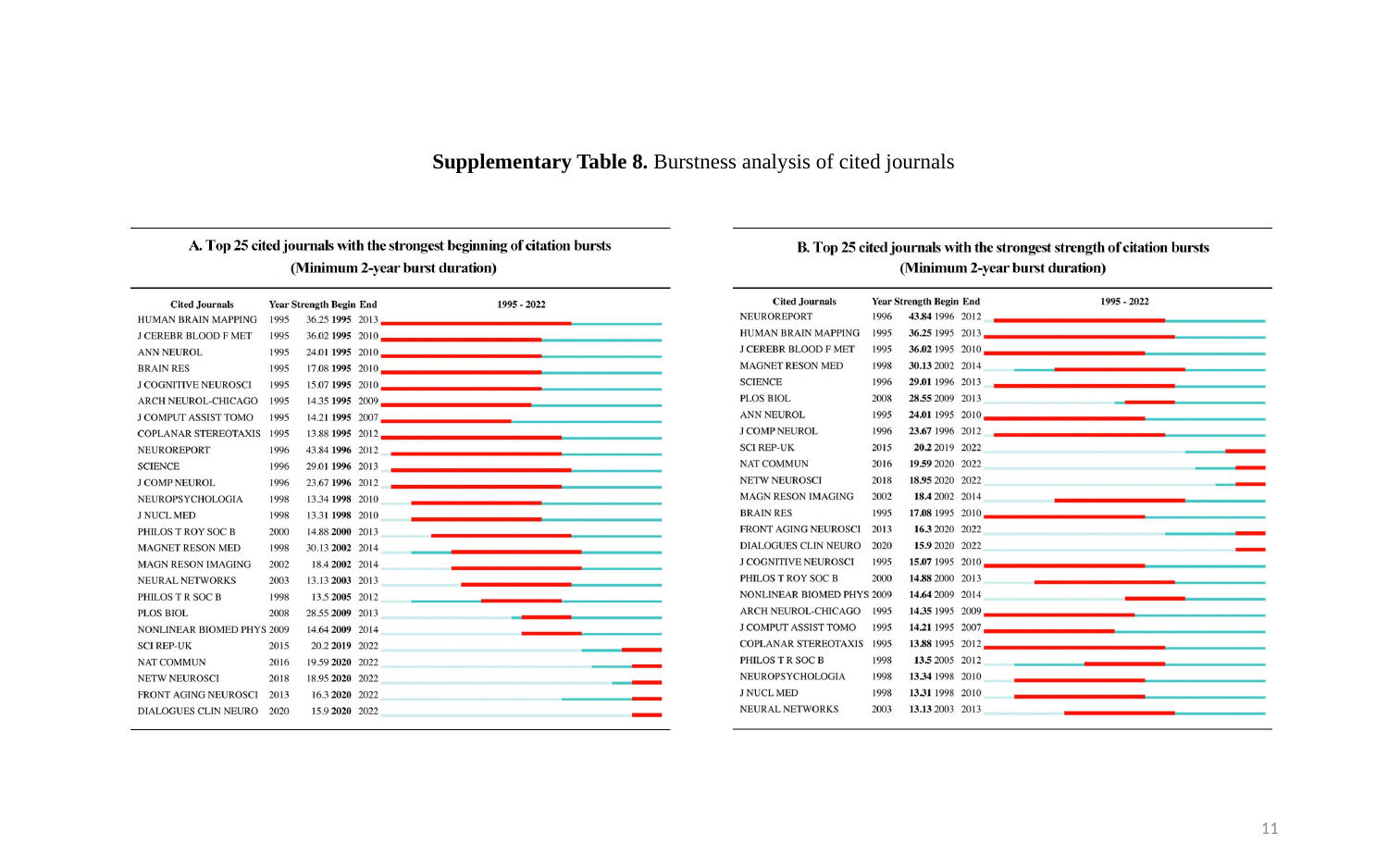

Supplementary Table 8. Burstness analysis of cited journals
11

## Slide 12
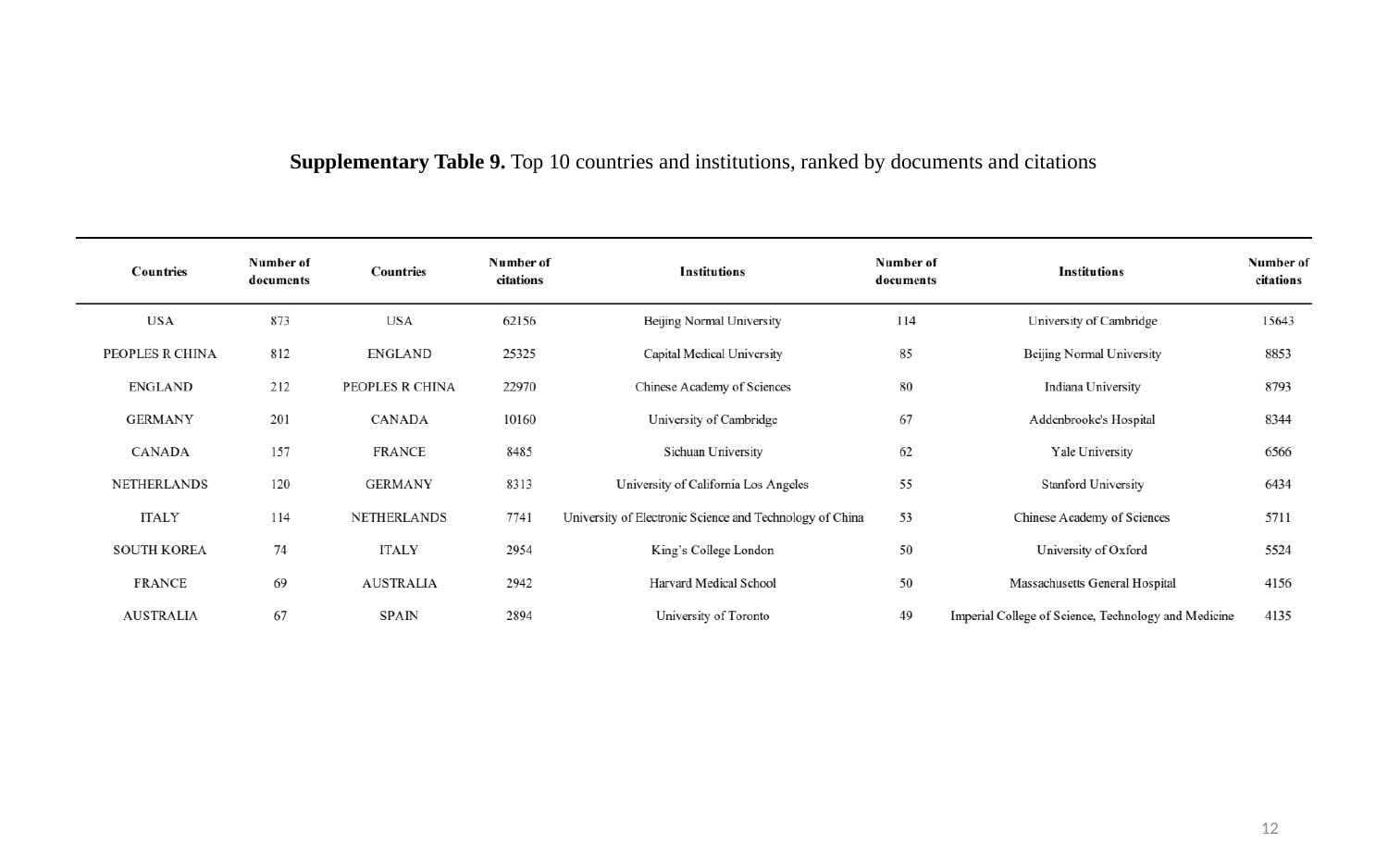

Supplementary Table 9. Top 10 countries and institutions, ranked by documents and citations
12
